# A Three-Year Look at the Phylogenetic Profile, Antimicrobial Resistance, and Associated Virulence Genes of Uropathogenic *Escherichia coli*

**DOI:** 10.3390/pathogens11060631

**Published:** 2022-05-30

**Authors:** Lorina I. Badger-Emeka, Naheed Kausar, Edric Estrella, Glenda Belgira Angeles

**Affiliations:** 1 Department of Biomedical Sciences, College of Medicine, King Faisal University, Al-Ahsa 31982, Saudi Arabia; nmurtaza@kfu.edu.sa (N.K.); gangenes@kfu.edu.sa (G.B.A.); 2 Department of Public Health, College of Applied Medical Sciences, King Faisal University, Al-Ahsa 31982, Saudi Arabia; eestrella@kfu.edu.sa

**Keywords:** uropathogenic, *Escherichia coli*, MDR, phylogroup, multidrug resistant, ESBL, virulence genes

## Abstract

Uropathogenic *Escherichia coli* is the most common cause of urinary tract infections, resulting in about 150 million reported annual cases. With multidrug resistance on the rise and the need for global and region surveillance, this investigation looks at the UPEC isolates collected for a 3-year period, with a view of ascertaining their antimicrobial susceptibility patterns and associated virulence determinants. The identification of bacteria isolates, antimicrobial susceptibility, and extended-spectrum beta-lactamases (ESBLs) production was determined with a Vitek 2 Compact Automated System (BioMerieux, Marcy L’Etoile, France). ESBLs were confirmed by the combined disc test (CDT) and basic biochemical test. The isolates were distributed into A (11%), B1 (6%), B2 (62.4%), and D (20.6%). Resistance to the penicillin group was high, between 88% and 100%. Additionally, resistance was high to cephalosporins (100%) in 2017 and 2018. The isolates were all sensitive to tigecycline, while resistance against imipenem and meropenem was low, at 4–12% in 2017 and 2018 and 0% in 2019. The results also showed that ESBL isolates were seen in 2017 and 2018. They were confirmed positive to CTX/CLA (88.5%) and CAZ/CLA (85%). By 2019, the number of resistant isolates reduced, showing only 4% ESBL isolates. Two virulence genes, *fim*H (46%) and *pap*E/F (15%), were detected among the isolates by PCR. In conclusion, this study found that phylogroups B2 and D carried the most virulence genes as well as MDR and ESBL characteristics, suggesting the UPEC strains to be extraintestinal pathogens responsible for UTIs.

## 1. Introduction

Urinary tract infections (UTIs) place a huge annual financial burden on healthcare systems globally. An estimated 150 million cases are reported globally on an annual basis [1]. It is estimated that there are about seven million hospital visits, of which one million are admitted to emergency units/departments in the USA annually [2,3], impacting patients’ quality of life [4]. Generally, UTIs can be categorized as either community-associated (CAUTIs) or hospital-acquired (HAUTIs) infections [5]. In Saudi Arabia, the incidences are high and are said to be the most prevalent cause of infections [6], predominantly so among women [7]. Uropathogenic *Escherichia coli* (UPEC) are highly versatile pathogens capable of both commensal existence in the human GIT as well as becoming opportunistic pathogens in the urinary tract [8]. Of UTI visits to hospitals, 14.6% were to emergency departments, reported in Riyadh, Saudi Arabia during a quarter of one year by adults and elderly patients [9]. This percentage was higher than that of an earlier report in the same region [10]. Additionally, higher frequencies of UTIs have been consistently reported among women patients in Saudi Arabia compared to male patients, the reasons for which are attributed to anatomical and physiological differences [9]. A report by the General Authority for Statistics, Saudi Arabia [11] placed the population of women at about 8.5 million in January 2016. Of these, there were an estimated 880,000 women in that year who visited primary care due to UTIs, with half of that number (440,000) being to emergency departments. Though frequencies of UTIs in women are indicated to decrease with age while increasing in adult males [12], there has generally been a financial burden in the management of UTIs in Saudi Arabia, similar to that found in global reports [9]. Treatment has become more costly with the rise in antimicrobial resistance by bacterial isolates and clinicians being faced with limited options, particularly with ESBL-producing pathogens.

A high prevalence of ESBL-MDR *E. coli* strains has been reported [13,14,15], with a recent study confirming *E. coli* as the most common cause [16] of UTIs. The prevalence of ESBL carriage by MDR UTI clinical isolates has been reported [16,17], and this suggests additional therapeutic implications as they are resistant to a wide range of antibiotics [16].

While there are several bacterial pathogens that cause UTIs [9,16], those originating from *Escherichia coli* form the majority. Sometimes, the symbiotic relationship is compromised, and the violation of the gastrointestinal barriers could lead to a wide range of infections. On the other hand, there are pathogenic strains that are classified as either enteric or extraintestinal *E. coli* (ExPEC) [18]. Uropathogenic *E. coli* (UPEC) is one of two pathotypes of ExPEC, a strain that exists in the gut and can disseminate to other parts of the human anatomy, initiating disease [18]. Generally, urinary tract infections are caused by uropathogenic *E. coli* [19] with strains that are marked by diversity in mobility and metabolism, and bacterial behaviors that predict pathogenicity rather than the carriage of any specified sets of genes [19]. Virulence factors play critical roles throughout the process of invasion, colonization, and bacterial multiplication, and thereby continue to gain attention from researchers globally [11,12,13,20,21,22], particularly in this era of difficult-to-treat bacterial infections. However, differences in virulence phenotypes and host susceptibility determine the associated risk of UTIs [19]. Additionally, antimicrobial resistance is linked to reduced virulence [23], while phylogenetic diversity among *E. coli* isolates is further highlighted [24]. However, reports on virulence determinants are scarce in the region of the present study.

As with other bacterial pathogens, antimicrobial resistance by uropathogens is reportedly on a global increase [21,25]. Researchers reveal that there are limited options available for treatment [26,27,28,29], while recommending steps to help curtail antimicrobial resistance and the spread of resistant genes associated with them. Global and regional antimicrobial surveillance programs are one such step. Such programs have been instituted in regions of Saudi Arabia [9,25,30], where they update regional surveillance as recommended to curtail the rise in antimicrobial resistance. The literature is, however, silent on the region of the present investigation regarding virulence factors associated with bacterial infections and resistance. To bridge this gap in knowledge, the present report looks at the antimicrobial phenotypes of *Escherichia coli* isolates associated with urinary tract UPEC infections in Al-Ahsa, a town in the southeast of Saudi Arabia. This study also examines the antimicrobial susceptibility pattern of *E. coli* clinical isolates collected from urine samples between 2017 and 2019. Additionally, virulence genes associated with the isolates were ascertained to find any possible correlation between antimicrobial resistances. Therefore, the objective of this investigation was to examine the phylogenetic groups of UPEC isolates, their antimicrobial susceptibility pattern, and ESBL carriage among the isolates. In addition, associated virulence factors were also examined. The investigation provides an insight into a three-year antimicrobial resistance pattern.

## 2. Materials and Methods

### 2.1. Ethical Consideration

*E. coli* clinical isolates were those of routine hospital diagnoses, which served as part of patient care. They were obtained from the microbial bank of the medical microbiology laboratory in the College of Medicine of King Faisal University. Patients were not involved in the study.

### 2.2. Bacterial Isolates

A retrospective study was carried out on uropathogenic *E. coli* isolates collected between 2017 and 2019. Confirmed *E. coli* isolates had been inoculated into MicrobankTM ready-to-use tubes (Pro-lab Diagnostic, Round Rock, TX, USA) incorporated with treated beads and cryopreservative fluid by the manufacturers. The methods of preservation and bacterial retrieval were performed according to the guidelines provided by the manufacturers (https://www.pro-lab-direct.com/v/vspfiles/microbank/microbank-wwp-portfolio.pdf, accessed on 19 June 2021). A total of 170 bacteria strains were used for the antimicrobial susceptibility assay. The isolates were given codes beginning with letters followed by a number. Bacteria IDs were confirmed with a Vitek Compact 2 (BioMerieux, Marcy L’Etoile, France) using GN ID cards. Additionally, basic biochemical tests that included citrate and lactose fermenting in addition to urease and indole tests were carried out. However, 152 isolates were used for ESBLs assay, while 48 of them were used for molecular analysis.

### 2.3. Antimicrobial Susceptibility Assay, Detection and Confirmation of ESBLs

Vitek 2 AST cards were used to test the susceptibility of the isolates against the following antibiotics: amoxicillin, amoxicillin/clavulanic acid, piperacillin/tazobactam, imipenem (Imp), meropenem (Mer), ciprofloxacin (Cp), cephalotin (Kf), cefuroxime (Cxm), ceftriaxone (Cax), cefotaxime (Cft), cefazolin (Cfz), ceftazidime (Caz), cefepime (Pime), aztreonam (AZT), Augmentin (Aug), ampicillin (Amp), gentamicin (Gm), tigecycline (Tig), nitrofurantoin (Fd), and trimethoprim/sulfamethoxazole (Sxt). The production of extended-spectrum beta-lactamases (ESBLs) was detected by a Vitek 2 Automated System and confirmed by the combined disc test (CDT) as recommended by the CLSI [31], using cefotaxime (CTX) or ceftazidime (CAZ) combined with clavulanic acid (CLA) as inhibitors. Tests were carried out on Muller–Hinton agar incubated aerobically at 35 °C for 24 h. They were seeded with strains of UPEC isolates, discs of 30 µg cefotaxime (CTX), 30 µg ceftazidime (CAZ), combined with 10 µg clavulanic acid (CLA). The results were interpreted as recommended by the CLSI [32] based on resistance to a single test against cefotaxime and ceftazidime individually and separately in combination with clavulanate.

The following primers were used for the amplification and detection of the carriage of ESBL (tem, ctx, and shv) genes by PCR, with the resultant products being stained with ethidium bromide and visualized by agarose gel electrophoresis: TEM-F-AGATCAGTTGGGTGCACGAG, TEM-R CAGTGCTGCAATGATACCG; CTX-F-ATGTGCAGYACCAGTAARGTKATGGC, CTX-R-TGGGTRAARTARGTSACCAGAAYCAGCGG; and SHV-F-GGGTTATTCTTATTTGTCGC, SHV-R-TTAGCGTTGCCAGTGCTC.

### 2.4. Hemolysin Production and Cell Surface Hydrophobicity Test

The isolates were initially screened for hemolysin production as described earlier [33] by culturing them overnight on 5% blood agar at 37 °C to detect hemolysis by the isolates. Additionally, the salt aggregation test (SAT) was used to determine cell surface hydrophobicity according to methods described earlier [33]. A bacterial suspension of 10 μL was prepared in a phosphate buffer and mixed with an ammonium sulphate solution with a molarity that ranged from 0.3125 to 0.5 m on a glass slide. SAT values were interpreted as described earlier [34].

### 2.5. DNA Extraction and Determination of the Phylogenetic Grouping of the Isolates

A Qiagen DNA extraction kit was used to extract the genomic DNA of the isolates according to the guidelines of the manufacturer. Briefly, a final volume of 50 μL, made up of 25 μL of the master mixture, 2 μL of each of the primers, and 100 ng of the DNA template, was used for PCR amplification. The cycling program was performed in AB Applied Biosystems thermal cycler (Foster City, California 94404, USA) and consisted of 30 cycles of 94 °C for 1 min, 62 °C for 45 s, and 72 °C for 1 min, The resultant product was stained with 0.5 µg/mL of ethidium bromide and resolved by gel electrophoresis on 1% agarose gel.

The isolates were grouped phylogenetically by the PCR method described earlier [35], using *chu*A and *yja*A DNA markers in combination with *Tsp*E4.C2, an anonymous DNA fragment. The presence or absence of these three DNA fragments was used to group the isolates into the following: group A (*chu*A−, *Tsp*E4.C2−), group B1 (*chu*A+, *yja*A+), group B2 (*chu*A+, *yja*A−), and group D (*yja*A). The obtained groupings were categorized according to the profiles previously described [35], with the primers shown in Table 1.

### 2.6. Determination of Associated Virulence Factors of Strains’ UPEC Isolates

The assay of urovirulence genes in the isolates that were detected by multiplex PCR using the extracted DNA product of each isolate was amplified with specific virulence primers in Table 1. The manufacturer’s guidelines were used to constitute a multiplex reaction mix, “providing a final concentration of 3 mM MgCl_2_ (3 × 0.85 mL), 5× Q-Solution (1 × 2.0 mL), RNase-Free Water (2 × 1.7 mL)”. To the multiplex reaction, a mix of 1 µL of extracted DNA template was added to obtain 50 µL of the final reaction. Cycling conditions were also as recommended by the manufacturers: an initial heat activation of HotStar Tag DNA polymerase at 95 °C for 15 min; 35 cycles of 30 s denaturation at 94 °C and annealing for 90 s at 63 °C followed by extension at 72 °C for 90 s, with 10 min of the final extension at 72 °C (www.qiagen.com/HB-0453, accessed on 19 June 2021). The amplified PCR product was stained with 0.5 µg/mL ethidium bromide and resolved by gel electrophoresis on 2% agarose gel. The experiment was repeated twice.

### 2.7. Statistical Analysis

GraphPad Prism version 9.2.0 was used to analyze the data pertaining to the susceptibility of the antimicrobials and presented as percentages. A Z-test for the difference of two proportions was used for pairwise comparison between resistance versus resistance and intermediate versus intermediate percentages using Stata MP version 13 with significance taken at *p*-value < 0.05. In addition, a Chi-test for homogeneity of proportion was utilized to test significant differences across the various phylogenetic groups. A *p*-value of less than 0.01 was considered statistically significant.

## 3. Results

### 3.1. Demography and Source of Samples

**Figure 1:**
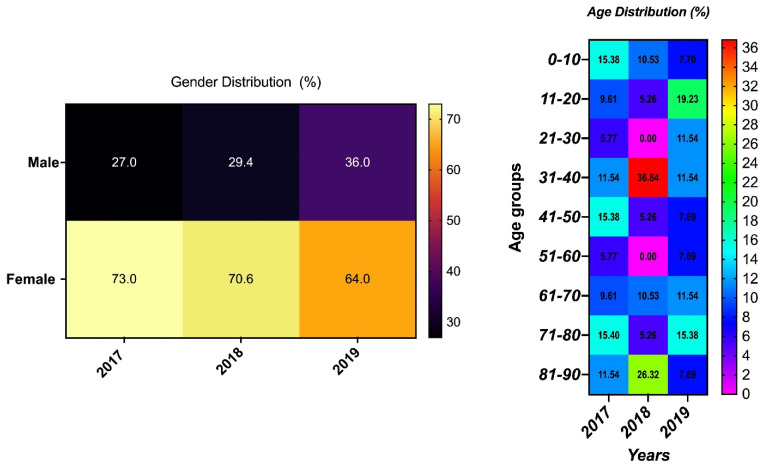
Demographic distribution of isolates’ sample sources with the year of isolation.

The UPEC isolates were from urine specimens that had been obtained from patients in wards, the out-patient department (OPD), the emergency room (ER), and the intensive care unit (ICU). Samples were more often from female patients than males, and this was consistently the case for the 3 years of observation. The age group ranged from less than 1 year to 90 years old; however, the age range of 31 to 40 years old was more prevalent compared to the other age groups (Figure 1).

### 3.2. Hemolysis, Cell Surface Hydrophobicity, and Phylogenetic Groups of the UPEC Isolates

Alpha (α) hemolysis on blood agar was detected in 48 (28.2%) of the initial 170 isolates while 64 (37.65%) of the isolates with visible bacterial clumping with an SAT of less than 1.25 were seen to be cell-surface hydrophobic [33,34]. Further investigation on this aspect was not carried out as they were basic initial microbial analysis.

Isolates were grouped into phylogroups A, BI, B2, and D (Table 2) by PCR gel electrophoresis results. Of the 170 UPEC strains, the majority (62.4%) were classified into the B2 phylogroup, next to which were those in the D phylogroup, which constituted 20.6% of the isolates. The least common were the A and B1 phylogroups which formed 11% and 6% of the UPEC isolates, respectively (Table 2).

### 3.3. Antimicrobial Susceptibility of the Isolates

The isolates were resistant to the penicillin group of antibiotics for the 3 years observed, showing high resistance to ampicillin (Aml) and amoxicillin (Am). Additionally, resistance to cephalotin (Kf), ceftazidime (Caz), ceftriaxone (Cax), and cefepime (Pime) was high (100%) in 2017 and remained at 100% for Kf with a slight decrease in resistance (94%) to Caz, PIME, and Cax (96%) in 2018. Resistance to the other cephalosporins (Cfz, Cft, and Cxm) was between 94% and 100% in the first 2 years observed, with a significant (*p* = 0.00) decrease to between 27% and 15% in the third year. Resistance to the carbapenems was low for all 3 years observed (4–12% in 2017 and 2018 to 0% in 2019). However, there was a high susceptibility of the isolates to tigecycline (100%) for the 3 years observed, but lower susceptibility was seen for tetracycline (20%, 35%, and 35%) for the 3-year duration. No specific pattern in the percentage of resistance to amikacin was seen for the duration observed (Table 3).

Among the fluoroquinolones (ciprofloxacin and levofloxacin), resistance remained between 70% and 90% among the isolates. For this group of antibiotics, there was no specific pattern of susceptibility during the 3 years observed. However, resistance by the isolates to trimethoprim/sulfamethoxazole (Sxt) increased nonsignificantly (*p* = 0.69, 0.09), from 73% in 2017, to 75% in 2018, and significantly (*p* = 0.04) to 85% in 2019 (Table 3).

### 3.4. Antimicrobial Pattern of ESBLs UPEC Strains

**Figure 2:**
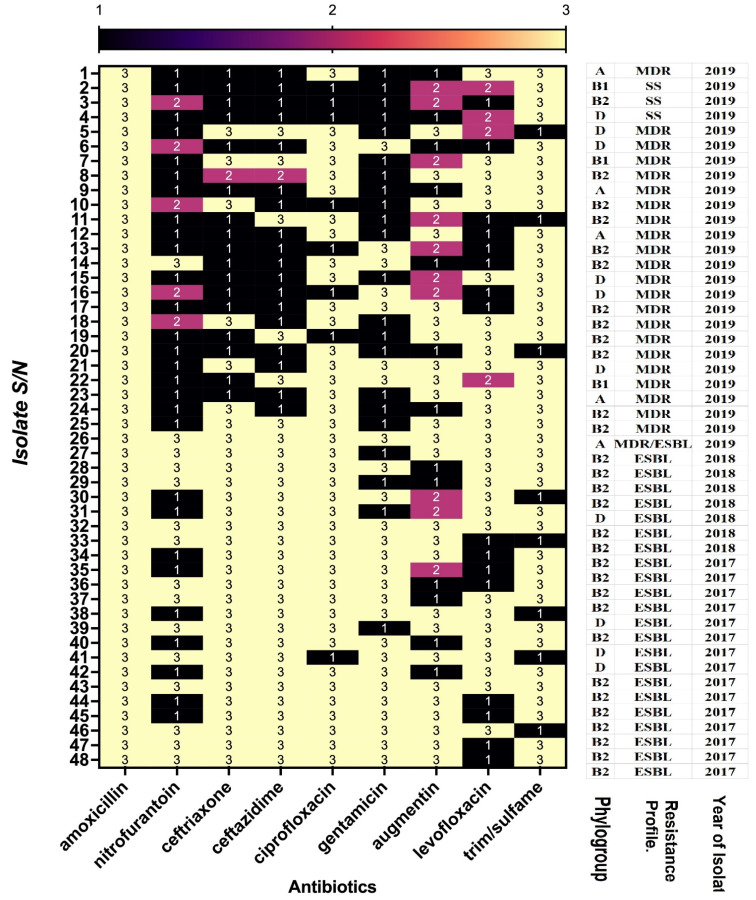
Comparison of antimicrobial resistance, isolate phylogenetic group, and the year of isolation: multidrug resistant (MDR); susceptible strain (SS); and extended-spectrum beta-lactamases (ESBL). Resistant (3); intermediate (2); sensitive (1). The figure shows the 48 UPEC isolates and their susceptibility to antimicrobials commonly used for treatment.

An overall look at the antimicrobial susceptibility of the isolates showed that those collected in 2019 with serial numbers (S/N) 1–26 were more sensitive to tested antibiotics compared to those with S/N 27–48 collected in 2018 and 2017 (Figure 2). All the 2017 and 2018 isolates are ESBLs, the majority of which were in the B2 and D phylogroups, while only AC 37 was an ESBL 2019 isolate (Figure 2).

UPEC–ESBL isolates in the first year of the investigation (2017) were confirmed positive to CTX/CLA (88.5%) and CAZ/CLA (85%); these percentages were reduced in 2018 (40% and 15%, respectively) and subsequently to 4% in 2019 (Table 4). Three investigated ESBL genes were detected among the isolates with varying proportions, more so with the 2017 isolates. CTX was detected in the majority of the isolates, irrespective of the year of sampling (Table 4). The results also showed some multiple carriage of antimicrobial-resistant genes among the isolates, while none of the investigated genes was detected in 15.4% and 2% of the samples in 2017 and 2018, respectively (Table 4).

### 3.5. Susceptibility to Antimicrobials According to Phylogenetic Group

All the phylogroups were resistant to the penicillins while exhibiting no specific pattern of susceptibility against the other tested antibiotics. There were, however, some variations among the groups as seen in the isolates in phylogroups A and D being significantly (*p* = 0.001) less resistant to Augmentin (Table 5) when compared to the other phylogroups. Additionally, no significant (*p* = 0.55) difference in the percentage numbers of UPEC isolates in phylogroups (A, B1, B2 and D) that were resistant to ceftriaxone were noted. Isolates in group A and D were significantly (*p* = 0.001) less resistant to the cephalosporin antibiotic ceftazidime. For ciprofloxacin, significant differences (*p* < 0.001) were seen in percentage resistance between phylogroup A and all others. However, comparing phylogroup B1 and D did not show any significant (*p* < 0.291) difference. For the second fluoroquinolone antibiotic (levofloxacin), differences seen in the phylogroups were not significant (*p* = 0.37). There were nonsignificant (0.15) differences in the susceptibility of all groups of the UPEC isolates to nitrofurantoin. Furthermore, while all of the B1 isolates were highly sensitive to nitrofurantoin, they were highly resistant to trimethoprim/sulfamethoxazole. Generally, susceptibility to tobramycin was low, with 94% (A), 75% (B1), 93% (B2), and 83% (D) resistance recorded among the phylogroups.

### 3.6. Associated Virulence Genes

Six virulence genes (*sfa*, *fim*H, *pap*E/F, *iron*N, *pap*A, and *hly*A) were investigated by PCR. Only two genes, *fim*H and *pap*E/F, were detected. A total of 46% of UPEC strains presented the *fim*H gene, and 15% of the isolates presented the *pap*E/F gene.

## 4. Discussion

This study describes the antimicrobial pattern and associated virulence genes in strains of *Escherichia coli* strains from urinary tract infections (UPECs) as seen over a period of three years in the Al-Ahsa region of Saudi Arabia. Samples were more often from females than males, and the highest amount came from the 30–40 year-old age group, findings that are consistent with those of a previous report [40]. Additionally, the isolates here were classified into A, B1, B2, and D phylogroups, with significantly more isolates in B2 (62.4%) and D (20.6%), suggesting the UPEC strains could be extraintestinal pathogens responsible for the UTIs [18]. The least common phylogroups were A and B1, which are considered as commensals. These findings are similar to those of other researchers [41,42,43].

The isolates in this investigation were MDR, being resistant to three or more groups of antimicrobials. For the penicillin group (Aml, Am), a high percentage of resistance (88–100%) was observed for the period of study. This is consistent with previous reports from other regions in the Kingdom [44,45], while in a recent report, resistance to ampicillin was low [16]. Nonetheless, the penicillin group of antibiotics might not be suitable in the management of UPEC urinary tract infections attributed to strains of *E. coli* in this locality. The 2019 clinical UPEC isolates were more susceptible to the tested drugs than the isolates from the preceding (2017, 2018) years were. This is consistent with reports on findings by researchers in the study region [46,47,48]. They reported that the implementation of antimicrobial stewardship by some hospitals in the Kingdom could also be a probable contributory factor [49]. In addition to this, there is the implemented regulation by the Saudi Arabia Ministry of Health (MOH) for dispensing nonprescribed antibiotics across the counter by community pharmacies [50]; this could also be taken into consideration. However, this trend is worth monitoring in antimicrobial surveillance when taking into consideration that the excessive use of broad-spectrum antibiotics has led to MDR–UPEC strains [51,52]. Generally, there is a high incidence of resistance to antibiotics in the treatment of UTI isolates in the region of this study [9,16,44], while it is thought that MDR seen in UTIs is on the rise [53]. Thus, the results here further highlight challenges posed by difficult-to-treat bacterial infections. The pattern of resistance to nitrofurantoin (Fd), seen for the three years observed, can, however, be considered high when compared with those of other reports [9,52,54,55,56]. However, the 8% recorded resistance to Fd in 2019 is similar to that of reported strains in hospitalized patients in England [57] and Bosnia-Herzegovina [58]. Thus, Fd could still serve as a first-line treatment in uncomplicated cases of UTIs caused by UPEC, as suggested earlier [52]. Other antibiotics recommended by the European Association of Urology in managing uncomplicated UTIs are fosfomycin and trimethoprim/sulfamethoxazole (SXT) [51], both of which were not suitable for the management of the UPEC isolates in this investigation. Similarly, for low susceptibility to SXT, a frontline antibiotic had been reported [59,60], with the suggested reasons for such resistance being the inappropriate wide use of these antibiotics [59]. Thus, it might not be suitable for managing infections resulting from MDR–UPEC isolates. Contrary to the present findings for fosfomycin are reports from Germany, Spain, and Belgium which found an antibiotic suitable for managing UPEC infections [55,61]. This, therefore, suggests variations that might be the result of regional differences, which should be taken into consideration in the management UPEC infections [51].

The UPEC isolates here were sensitive to tigecycline and exhibited low resistance to amikacin, imipenem, and meropenem. A recent report in Saudi Arabia listed amikacin and meropenem as the best antimicrobials in the management of UTIs [16]. These drugs are still the gold standard in the management of MDR–UPEC infections in this region of study. However, research findings vary, with reports stating that MDR–UPEC isolates are sensitive to amikacin on the one hand and resistant to carbapenem on the other [41]. Carbapenems are commonly recommended for the management of ESBL–UPEC infections according to Kot [52].

The highest percentage number of ESBL–UPEC strains was found among the 2017 isolates, findings that are similar to those of others in Saudi Arabia [44,62,63] as well as other regions of the world [41]. The ESBL–UPEC isolates in this research were predominant in the B2 phylogroup, with bla_CTX_ as the main amplified gene, and in some cases, multiple ESBL genes were detected, findings that agree with those of other researchers [41,64]. Here, the predominance of the bla_CTX_ gene, as well as the encountered coexistence with other ESBL genes, could give bacteria selective advantage against antibiotic pressure [41]. The prevalence of the bla_CTX_ gene has also been reported in different regions of the world [40,65,66,67].

There is also the carriage of virulence genes either chromosomally or extra-chromosomally by UPEC isolates, all of which are established during the course of an infection. Generally, the B2 and D phylogroups are stipulated to be the most virulent strains associated with UTIs [68], both of which were in the majority in this study. Only two of the six investigated virulence genes were detected in this study, of which the adhesion gene *fim*H was significantly more common than *pap*E/F. This might be considered, overall, as a low carriage of virulence genes when compared to findings by other researchers [68]. The reasons for the low carriage of virulence genes can be attributed to several reasons. First is the number of investigated genes compared to the vast number of those associated with strains of UPEC. Bearing in mind the genomic diversity of *E. coli*, the mobile genetic elements collectively determine pathotype and ecotype trait [69]. Numerous virulence elements result from different genes that are detectable by PCR [65,70,71]. Another reason that should be taken into consideration is the genetically diverse subset of extraintestinal pathogenic *E. coil* which cause UTIs and are reported to have no core sets of virulence factors [8]. There is also a functional redundancy which can lead to the expression of mixed bacterial populations. In addition, hemolysis as seen in the preliminary study is generally associated with cell lysis and considered a cytotoxic necrotizing factor [33]. In addition, the observed hydrophobicity in some of the strains here has been described as virulence mechanism [33].

However, due to possible gene mutations, PCR amplification could lead to not detecting virulence genes, while negative PCR results could therefore not be considered as being the absence of a targeted gene, a view suggested earlier [72]. Again, UPEC isolates are postulated to parade a high degree in the diverse possession of specific virulence genes on their pathogenicity island [18,73]. The course of infection, as well as the duration of infection, could also be a leading factor in the process of virulence, but this was not investigated here. There is a need for further investigation.

## 5. Conclusions

We have reported the phylogenetic groupings of UPEC isolates collected in the Al-Ahsa region in the southeast of Saudi Arabia for a three-year period. The majority of the isolates were MDR, with those collected before 2019 being less susceptible to recommended first-line antibiotics. In addition, the four phylogroups, A, B1, B2, and D, showed nonspecific patterns in their drug-resistant profiles. Interestingly, the 2019 antimicrobial susceptibility showed a better sensitivity pattern with the isolates of that year. A minimal number of virulence genes was detected among the UPEC isolates irrespective of the year of isolation. We therefore suggest further investigation, particularly on recurrent infections with UPEC.

## Figures and Tables

**Table 1:** Oligonucleotide sequences of primers and the molecular weight (bp) used for the research.

Genes	Primer Sequence	BP	Reference
*Chu*A	F-GACGAACCAACGGTCAGGAT R-TGCCGCCAGTACCAAAGACA	279	[35]
yjaA	F-TGAAGTGTCAGGAGACGCTG R-ATGGAGAATGCGTTCCTCAAC	211	[35]
*TspE*4C2	F-GAGTAATGTCGGGGCATTCA R-CGCGCCAACAAAGTATTACG	154	[35]
*fim*H	F: TGCAGAACGGATAAGCCGTGG R: GCAGTCACCTGCCCTCCGGTA	506	[36]
*Sfa* (*sfa*/*foc*)	F: CTCCGGAGAACTGGGTGCATCTTAC R: CGGAGGAGTAATTACAAACCTGGCA	410	[37]
*pap*A	F: ATGGCAGTGGTGTTTTGGTG R: CGTCCCACCATACGTGCTCTTC	720	[37]
*pap*E/F	F: GCAACAGCAACGCTGGTTGCATCAT R: AGAGAGAGCCACTCTTATACGGACA	336	[38]
*hly*A	F: AACAAGGATAAGCACTGTTCTGGCT R: ACCATATAAGCGGTCATTCCCGTCA	1170	[38]
*iro*N	F AAGTCAAAGCAGGGGTTGCCCG R-GACGCCGACATTAAGACGCAG	665	[39]

**Table 2:** Phylogenetic groups of the isolates and the distribution of the virulence genes.

S/N	Lab ID	Genes/Phylogenetic Group				Virulence Genes					
		*chu*A	*yja*A	*Tsp*E4.C2	Group	*sfa*	*fim*H	*pap*E/F	*iro*N	*pap*A	*hly*A
1	AC12	−		−	A	−	−	+	−	−	−
2	AC13	−		+	B1	−	−	−	−	−	−
3	AC14	+	+		B2	−	+	−	−	−	−
4	AC15	+	−		D	−	−	−	−	−	−
5	AC16	+	−		D	−	−	−	−	−	−
6	AC17	+	−		D	−	−	−	−	−	−
7	AC18	−		+	B1	−	−	−	−	−	−
8	AC19	+	+		B2	−	−	+	−	−	−
9	AC20	−		−	A	−	−	−	−		−
10	AC21	+	+		B2	−	−	+	−	−	−
11	AC22	+	+		B2	−	+	−	−	−	−
12	AC23	−		−	A	−	−	−	−	−	−
13	AC24	+	+		B2	−	+	−	−	−	−
14	AC25	+	+		B2	−	+	−	−	−	−
15	AC26	+	−		D	−	−	−	−	−	−
16	AC27	+	−		D	−	−	−	−	−	−
17	AC28	+	+		B2	−	+	−	−	−	−
18	AC29	+	+		B2	−	−	+	−	−	−
19	AC30	+	+		B2	−	+	−	−	−	−
20	AC31	+	+		B2	−	+	−	−	−	−
21	AC32	+	−		D	−	+	−	−	−	−
22	AC33	−		+	B1	−	−	−	−	−	−
23	AC34	−		−	A	−	−	−	−	−	−
24	AC35	+	+		B2	−	+	−	−	−	−
25	AC36	+	+		B2	−	+	−	−	−	−
26	AC37	−		−	A	−	−	−	−	−	−
27	LAB07	+	+		B2	−	−	+	−	−	−
28	LAB31	+	+		B2	−	−	−	−	−	−
29	LAB40	+	+		B2	−	+	−	−	−	−
30	LAB63	+	+		B2	−	+	−	−	−	−
31	LAB71	+	−		D	−	−	−	−	−	−
32	LAB87	+	+		B2	−	+	−	−	−	−
33	LAB91	+	+		B2	−	−	+	−	−	−
34	LAB114	+	+		B2	−	−	−	−	−	−
35	LAB119	+	+		B2	−	+	−	−	−	−
36	LAB122	+	+		B2	−	−	−	−	−	−
37	LAB124	+	+		B2	−	−	−	−	−	−
38	LAB130	+	−		D	−	+	−	−	−	−
39	LAB140	+	+		B2	−	−	+	−	−	−
40	LAB149	+	−		D	−	+	−	−	−	−
41	LAB155	+	−		D	−	+	−	−	−	−
42	LAB158	+	+		B2	−	−	−	−	−	−
43	LAB168	+	+		B2	−	+	−	−	−	−
44	LAB186	+	+		B2	−	−	−	−	−	−
45	LAB188	+	+		B2	−	+	−	−	−	−
46	LAB193	+	+		B2	−	+	−	−	−	−
47	LAB194	+	+		B2	−	+	−	−	−	−
48	LAB195	+	+		B2	−	+	−	−	−	−

Virulence genes: *sfa* (0/48); *fim*H (22/48); *pap*E/F (7/48); *iro*N (0/48); *pap*A (0/48); and *hly*A (0/48). Detection (+); absence (−).

**Table 3:** Percentage (%) of antimicrobial susceptibility of clinical strains of *E. coli* urine from urine samples for a period of 3 years.

Antimicrobial Classes	Antimicrobials	Year of Isolation						% R Pairwise Comparison (*p* Value)			% I Pairwise Comparison (*p* Value)		
		2017		2018		2019		2017 vs. 2018	2017 vs. 2019	2018 vs. 2019	2017 vs. 2018	2017 vs. 2019	2018 vs. 2019
		% R	% I	% R	% I	% R	% I						
Penicillins	Aml	np	np	88	0	100	0	na	na	<0.01	na	na	-
	Am	100	0	100	0	100	0	-	-	-	-	-	-
	Aug	73.08	0	64.7	0	46	27	0.20	<0.01	0.01	-	<0.01	<0.01
	Ptz	np	np	35.3	0	23	4	na	na	0.06	na	na	0.04
Cephalosporins	Cfz	100	0	100	0	np	np	-	na	na	-	na	na
	Cft	100	0	94.1	0	30.8	7.7	0.01	<0.01	<0.01	-	<0.01	<0.01
	Cxm	100	0	100	0	89	0	-	<0.01	<0.01	-	-	-
	Kf	100	0	100	0	57.7	27	-	<0.01	<0.01	-	<0.01	<0.01
	Fox	np	np	np	np	38.46	0	na	na	na	na	na	na
	Caz	100	0	94.1	0	26.92	0	0.01	<0.01	<0.01	-	-	-
	Cax	100	0	96	0	27	0	0.04	<0.01	<0.01	-	-	-
	Pime	100	0	94.1	0	15.4	0	0.01	<0.01	<0.01	-	-	-
Carbapenems	Imi	4.6	0	12	0	0	8	0.06	0.03	<0.01	-	<0.01	<0.01
	Mer	2	0	0	0	4	4	0.16	0.41	0.04	-	0.04	0.04
Aminoglycosides	Amk	11	2	23.5	0	4	4	0.02	0.06	<0.01	0.16	0.41	0.04
	Gm	66	0	53	0	27	0	0.06	<0.01	<0.01	-	-	-
Fluroquinolones	Cp	85.7	0	70.5	0	73	0	0.01	0.03	0.69	-	-	-
	Levo	81.4	0	76.5	0	90	0	0.40	0.08	0.01	-	-	-
Others	Tig	0	0	0	0	0	0	-	-	-	-	-	-
	Fd	35	0	59	0	8	19	<0.01	<0.01	<0.01	-	<0.01	<0.01
	Sxt	72.55	0	75	0	84.6	0	0.69	0.04	0.09	-	-	-
	Azt	73	0	100	0	75	0	<0.01	0.75	<0.01	-	-	-
	C	25	7	7	25	np	np	<0.01	na	na	<0.01	na	na
	Pip	100	0	100	0	np	np	-	na	na	-	na	na
	Te	79.6	0	64.7	0	65	0	0.02	0.02	0.96	-	-	-

Table shows Pairwise comparisons of the % R in 2017 vs. 2018, 2017 vs. 2019, and 2018 vs. 2019, performed using a z-test for the difference of two proportions; Similarly, % I in 2017 vs. 2018, 2017 vs. 2019, and 2018 vs. 2019 were compared utilizing the same statistical test; A *p*-value of less than 0.05 is considered statistically significant. Resistance (R), Intermediate (I), versus (vs). Ampicillin (Aml), amoxicillin (Am), amoxicillin/clavulanic acid (Aug), piperacillin/tazobactam (Ptz), cephalotin (Kf), cefoxitin (Fox), ceftazidime (Caz), Ceftriaxone (Cax), cefepime (Pime), imipenem (Imp), meropenem (Mer), amikacin (Amk), gentamicin (Gm), ciprofloxacin (Cp), tigecycline (Tig), nitrofurantoin (Fd), trimethoprim/sulfamethoxazole (Sxt), aztreonam (Azt), Cefazolin (Cfz}, Cefotaxime (Cft), cefoxitin (Ctt), cefuroxime (Cxm), Chloramphenicol (C), levofloxacin (Levo), Piperacillin (Pip), and Tetracycline (TE); not performed (np); not applicable (na); not calculable (-).

**Table 4:** ESBLs and associated resistant determinant genes.

ESBL Test	Year of Isolation		
	2017	2018	2019
	N = 52 (%)	N = 50 (%)	N = 50 (%)
CTX/CLA	46 (88.5)	20 (40)	2 (4)
CAZ/CLA	44 (85)	15 (30)	1 (2)
Negative for CTX/CAZ/CLA	6 (11.5)	0 (0)	0 (0)
Associated extended-spectrum beta-lactamases (ESBL) genes			
ctx	44 (85)	15 (30)	0 (0)
shv	13 (25)	10 (20)	0 (0)
tem	29 (56)	6 (12)	0 (0)
ctx/shv	13 (25)	3 (6)	1 (2)
ctx/tem	18 (34.6)	5 (10)	0 (0)
shv/tem	1 (2)	0 (0)	0 (0)
ctx/shv/tem	8 (15.4)	3 (6)	1 (2)
None	8 (15.4)	1 (2)	0 (0)

Results are presented as percentages (%), with N = Number.

**Table 5:** Antimicrobial susceptibility distribution among the phylogenetic groups.

Antibiotics	Phylogroups, N (%)								Comparison of % R across Phylogroups *X*^2^, *p* Value
	A N = 18		B1 N = 11		B2 N = 106		D N = 35		
	R %	I %	R %	I %	R %	I %	R %	I %	
Amoxicillin	18 (100)	0 (0)	11 (100)	0 (0)	106 (100)	0 (0)	35 (100)	0 (0)	na
Augmentin	11 (60)	0 (0)	4 (36)	7 (64)	64 (60.4)	14 (13.2)	14 (40)	11 (31)	58.32, <0.001
Ceftriaxone	14 (78)	4 (22)	8 (73)	0 (0)	74 (70)	6 (6)	21 (60)	2 (6)	2.07, 0.558
Ceftazidime	0 (0)	0 (0)	8 (73)	0 (0)	67 (63)	7 (7)	14 (40)	0 (0)	28.76, <0.001
Ciprofloxacin	18 (100)	0 (0)	7 (64)	0 (0)	82 (77)	0 (0)	25 (71)	0 (0)	0.001
Gentamicin	4 (22)	0 (0)	8 (73)	0 (0)	53 (50)	0 (0)	25 (71)	0 (0)	13.72, 0.003
Nitrofurantoin	4 (22)	0 (0)	0 (0)	0 (0)	25 (24)	11 (10)	11 (31)	0 (0)	0.15
Levofloxacin	11 (61)	0 (0)	7 (64)	4 (36)	53 (50)	14 (13)	14 (40)	14 (40)	3.11, 0.375
Trimethoprim/ Sulfamethoxazole	11 (61)	0 (0)	11 (100)	0 (0)	96 (91)	0 (0)	25 (71)	0 (0)	0.01
Tobramycin	17 (94)	0 (0)	8 (73)	0 (0)	98 (93)	0 (0)	29 (83)	0 (0)	0.01

Number (N), Percentage (%), Resistant (R), Intermediate (I), not applicable (na). A *p*-value of less than 0.01 is considered statistically significant. However, StatPac version 4 was used to calculate significant differences for ciprofloxacin, trimethoprim/sulfamethoxazole, and tobramycin.

## Data Availability

The data are present in the paper, but should the need arise, the corresponding author can also be contacted.
